# Supplementary material for: Principal neuron diversity in the murine lateral superior olive supports multiple sound localization strategies and segregation of information in higher processing centers
Source: Commun Biol. 2023 Apr 19;6:432. doi: 10.1038/s42003-023-04802-5 (PMC10115857; doi:10.1038/s42003-023-04802-5)
Supplement: Supplementary file 2 — Supplementary Information [file 42003_2023_4802_MOESM2_ESM.pdf]

Supplemental information

Title: Principal neuron diversity in the murine lateral superior olive supports multiple sound localization strategies and segregation of information in higher processing centers

Authors and affiliations

Hariprakash Haragopal[1], *Bradley D. Winters[1,2]

[1]Department of Anatomy and Neurobiology and Hearing Research Group, Northeast Ohio Medical University, Rootstown, OH, United States; [2]Brain Health Research Institute, Kent State University, Kent, OH, United States

Corresponding author and lead contact *

Bradley D. Winters: Northeast Ohio Medical University, Department of Anatomy and Neurobiology and Hearing Research Group, 4209 State Route 44, Rootstown, OH 44272, United States, bwinters@neomed.edu.

Supplemental Figure S1

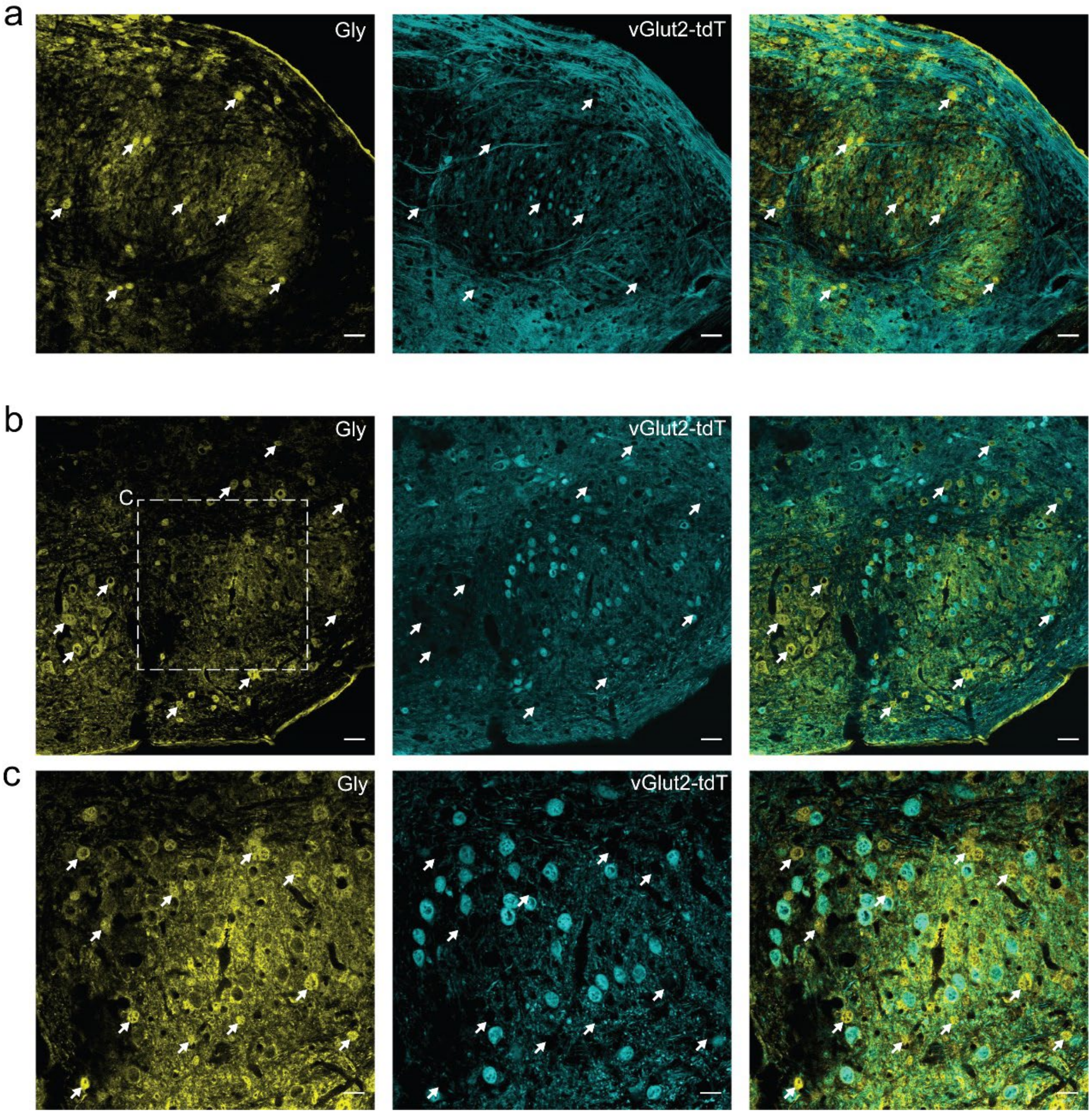

Figure S1: Cells with high glycine immunoreactivity are vGlut2 negative. a. Maximum projection of z-stacked images acquired using Olympus laser scanning confocal microscope at 20X. Yellow is immunoreactivity for glycine. Blue is tdTomato expressed under the control of vGlut2. Arrows indicate putative inhibitory cells with high glycine immunoreactivity. Scale 40 µm. **b.** As A in tissue from a different mouse. **c.** Outlined region in B imaged at 40X. Scale 20 µm.

Since glycinergic neurons are thought to maintain high cytosolic glycine, we used immunocytochemistry for glycine in glutaraldehyde fixed tissue from two vGlut2-tdT mice (see methods) to evaluate overlap between putative inhibitory and excitatory neurons in the superior olivary complex using our mouse model. Glycine immunoreactivity was not explicitly quantifiable

as all cells have some of this ubiquitous amino acid, however, we found that cells with high relative glycine immunoreactivity (arrows) did not have vGlut2 driven tdTomato expression. Methods: Mice were perfused with PBS for 4 min at 5 ml/min then 2% glutaraldehyde and 1% paraformaldehyde buffered in PBS for 5 min then post-fixed for 60 min and cryoprotected in 15% then 30% sucrose overnight. Tissue was then coated in OCT and frozen on dry ice then sliced on a cryostat at 40 µm. Slices were incubated in fresh 1% $NaBH_4$ for 30 minutes to reduce autofluorescence from glutaraldehyde fixation then rinsed 3 X 10 min in PBS on a shaker. Slices were blocked and permeabilized in 2% bovine serum albumin (BSA) with 5% normal donkey serum (NDS) in 0.3% Triton X-100 and 0.1% Tween 20 in PBS for 2 h at RT on a shaker. Slices were incubated in primary antibody solution containing primary antibodies (tdTomato: Sicgen AB8181-200, goat polyclonal, 1:250; Glycine: Millipore-Sigma AB139, rabbit polyclonal, RRID:AB_90582, 1:500), 2% BSA, 2% NDS and 0.2% Triton X-100 in PBS for overnight at 4 °C. Slices were incubated in secondary antibody solution containing secondary antibodies (Thermofisher, Donkey anti-Goat alexa 555, 1:200; Donkey anti-rabbit alexa 647, 1:200), 2% BSA, 2% NDS and 0.2% Triton X-100 in PBS for 2 h at RT on a shaker. Slices were mounted on super-frost slides using PBS, allowed to air dry for ~30 min, then coversliped with DAPI mounting media (Southern Biotech).

Supplemental Figure S2

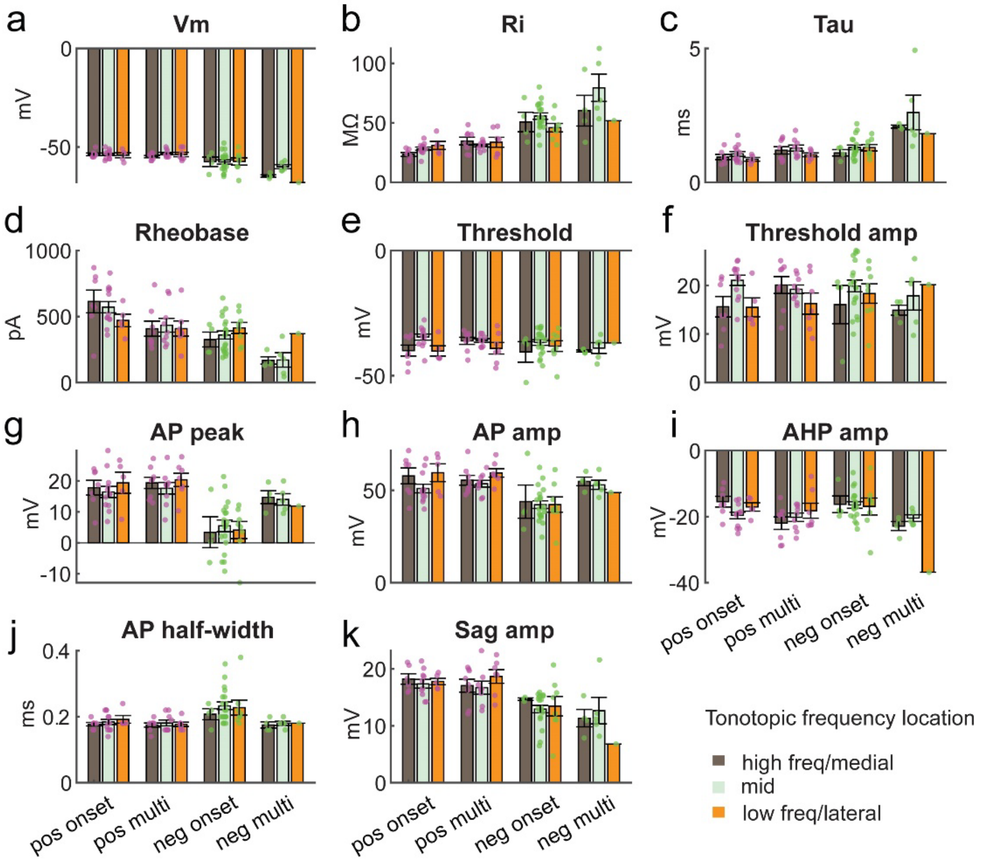

Figure S2: Analysis of electrophysiological parameters and tonotopy. Mean ±SEM of electrophysiological parameters for cell groups broken down by tonotopic locations. Data points in magenta: excitatory cells, green: inhibitory cells. There was only one inhibitory multi-spiking cell in the lateral/low frequency region. **a.** Resting membrane potential. **b.** Input resistance. **c.** Membrane time constants. **d.** Rheobase.**, e.** Action potential (AP) threshold. **f.** AP threshold amplitude. **g.** AP peak voltage. **h.** AP peak amplitude. **i.** Afterhyperpolarization amplitude. **j.** AP half-width. **k**. Sag potentials

We performed three-way ANOVA in MATLAB with independent variables transmitter type, firing type, and tonotopic location with the dependent variables shown. See tables below.

Table S1: Three-way ANOVA results for electrophysiological parameters with tonotopy and transmitter type. Analysis of data shown in Figure S2. Bold values are significant at an alpha value of 0.05.

	Tonotopy				**Transmitter x tonotopy**				Firing x tonotopy			
Table S1	df1	df2	F	p	df1	df2	F	p	df1	df2	F	p
RMP	2	79	1.3024	0.2776	2	79	0.2982	0.7430	2	79	1.4869	0.2323
**Ri**	2	79	2.3362	0.1033	2	79	3.4314	**0.0372**	2	79	0.4001	0.6716
tau	2	77	2.1075	0.1285	2	77	0.4100	0.6651	2	77	0.4566	0.6351
rheobase	2	79	0.2629	0.7695	2	79	2.6089	0.0799	2	79	0.9903	0.3760
threshold	2	79	2.4331	0.0943	2	79	1.1918	0.3091	2	79	1.4251	0.2466
threshold amplitude	2	79	2.5116	0.0876	2	79	1.2801	0.2837	2	79	1.3526	0.2645
AP peak	2	79	0.0706	0.9318	2	79	0.6683	0.5155	2	79	0.0795	0.9237

AP peak amplitude	2	79	1.0198	0.3654	2	79	1.3329	0.2696	2	79	0.2379	0.7889
AHP amplitude	2	79	0.1954	0.8229	2	79	2.6860	0.0744	2	79	2.2720	0.1098
AP halfwidth	2	79	0.7035	0.4979	2	79	0.1789	0.8365	2	79	0.1984	0.8204
Sag potential	2	74	0.1794	0.8362	2	74	0.5036	0.6064	2	74	0.4132	0.6630

Table S2: Three-way ANOVA results for electrophysiological parameters with transmitter type and firing type. Analysis of data shown in Figure S2. Bold values are significant at an alpha value of 0.05.

	**Transmitter**				**Firing**				**Transmitter x firing**			
Table S2	df1	df2	F	p	df1	df2	F	p	df1	df2	F	p
RMP	1	79	55.3764	**0.00**	1	79	10.8661	**0.0015**	1	79	9.4289	**0.0029**
Ri	1	79	79.9849	**0.00**	1	79	11.6862	**0.0010**	1	79	3.7639	0.0559
tau	1	77	31.7052	**0.00**	1	77	26.4842	**0.0000**	1	77	14.7702	**0.0002**
rheobase	1	79	24.2138	**0.00**	1	79	13.3810	**0.0005**	1	79	0.0197	0.8888
threshold	1	79	0.8841	0.35	1	79	0.1298	0.7196	1	79	0.4150	0.5213
threshold amplitude	1	79	0.1257	0.72	1	79	0.0417	0.8387	1	79	0.4786	0.4911
AP peak	1	79	29.5751	**0.00**	1	79	9.0418	**0.0035**	1	79	5.9318	**0.0171**
AP peak amplitude	1	79	12.5907	**0.00**	1	79	4.1889	**0.0440**	1	79	4.6424	**0.0342**
AHP amplitude	1	79	1.8391	0.18	1	79	19.6829	**0.0000**	1	79	4.4062	**0.0390**
AP halfwidth	1	79	4.3534	**0.04**	1	79	8.1654	**0.0055**	1	79	5.5102	**0.0214**
**Sag potential**	1	74	39.4182	**0.00**	1	74	2.5064	0.1177	1	74	1.1223	0.2929

Supplemental Figure S3

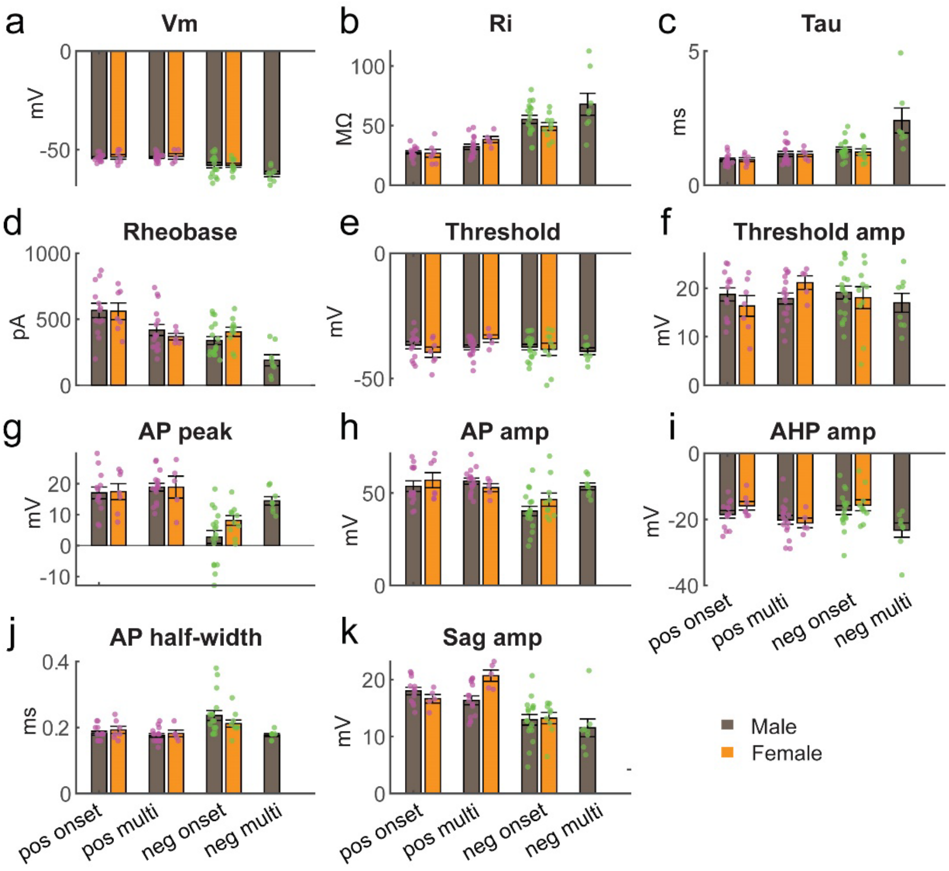

Figure S3: Analysis of electrophysiological parameters and sex. Mean ±SEM of electrophysiological parameters for cell groups broken down by sex. Data points in magenta: excitatory cells, green: inhibitory cells. There were no female inhibitory multi-spiking cells in the dataset. **a.** Resting membrane potential. **b.** Input resistance. **c.** Membrane time constant. **d.** Rheobase.**, e.** Action potential (AP) threshold. **f.** AP threshold amplitude. **g.** AP peak voltage. **h.** AP peak amplitude. **i.** Afterhyperpolarization amplitude. **j.** AP half-width. **k**. Sag potential.

We performed three-way ANOVA in MATLAB with independent variables transmitter type, firing type, and sex with the dependent variables shown. See tables below.

Table S3: Three-way ANOVA results for electrophysiological parameters with sex and transmitter type. Analysis of data shown in Figure S3. Bold values are significant at an alpha value of 0.05.

Table S3	Sex				Transmitter x sex				**Firing x sex**			
	df1	df2	F	p	df1	df2	F	p	df1	df2	F	p
RMP	1	69	0.0647	0.7999	1	69	0.0307	0.8614	1	69	0.0094	0.9231
Ri	1	69	0.0004	0.9835	1	69	0.4306	0.5139	1	69	0.6392	0.4268
tau	1	67	0.1182	0.7320	1	67	0.1371	0.7124	1	67	0.0024	0.9610
rheobase	1	69	0.0302	0.8625	1	69	0.6402	0.4264	1	69	0.1872	0.6666
threshold	1	69	0.6287	0.4305	1	69	0.3998	0.5293	1	69	3.1934	0.0783

threshold amplitude	1	69	0.4094	0.5244	1	69	0.1654	0.6855	1	69	2.3340	0.1311
AP peak	1	69	1.5899	0.2116	1	69	1.5147	0.2226	1	69	0.0074	0.9318
AP peak amplitude	1	69	0.2080	0.6498	1	69	0.2745	0.6020	1	69	1.1116	0.2954
AHP amplitude	1	69	0.0295	0.8640	1	69	0.1317	0.7178	1	69	1.0208	0.3159
AP halfwidth	1	69	0.6295	0.4303	1	69	2.3450	0.1303	1	69	0.0255	0.8736
**Sag potential**	1	64	4.7838	**0.0324**	1	64	0.5853	0.4470	1	64	5.7809	**0.0191**

Table S4: Three-way ANOVA results for electrophysiological parameters with transmitter type and firing type. Analysis of data shown in Figure S3. Bold values are significant at an alpha value of 0.05.

	**Transmitter**				**Firing**				**Transmitter x firing**			
Table S4	df1	df2	F	p	df1	df2	F	p	df1	df2	F	p
**RMP**	1	69	29.4792	**0.00**	1	69	2.6655	0.1071	1	69	5.1944	**0.0258**
**Ri**	1	69	50.4759	**0.00**	1	69	7.1583	**0.0093**	1	69	1.3054	0.2572
**tau**	1	67	22.5576	**0.00**	1	67	13.2975	**0.0005**	1	67	9.3566	**0.0032**
**rheobase**	1	69	15.7985	**0.00**	1	69	10.1896	**0.0021**	1	69	0.0000	0.9958
threshold	1	69	0.0138	0.91	1	69	1.0311	0.3134	1	69	0.0681	0.7948
threshold amplitude	1	69	0.0573	0.81	1	69	0.4721	0.4943	1	69	0.1780	0.6744
**AP peak**	1	69	10.2533	**0.00**	1	69	7.6068	**0.0074**	1	69	7.0018	**0.0101**
**AP peak amplitude**	1	69	4.8930	**0.03**	1	69	1.9522	0.1668	1	69	4.1091	**0.0465**
**AHP amplitude**	1	69	0.7873	0.38	1	69	9.3893	**0.0031**	1	69	2.3292	0.1315
**AP halfwidth**	1	69	0.7313	0.40	1	69	7.0063	**0.0101**	1	69	6.3658	**0.0139**
**Sag potential**	1	64	13.1172	**0.00**	1	64	1.1721	0.2830	1	64	0.0172	0.8961

Supplemental Figure S4

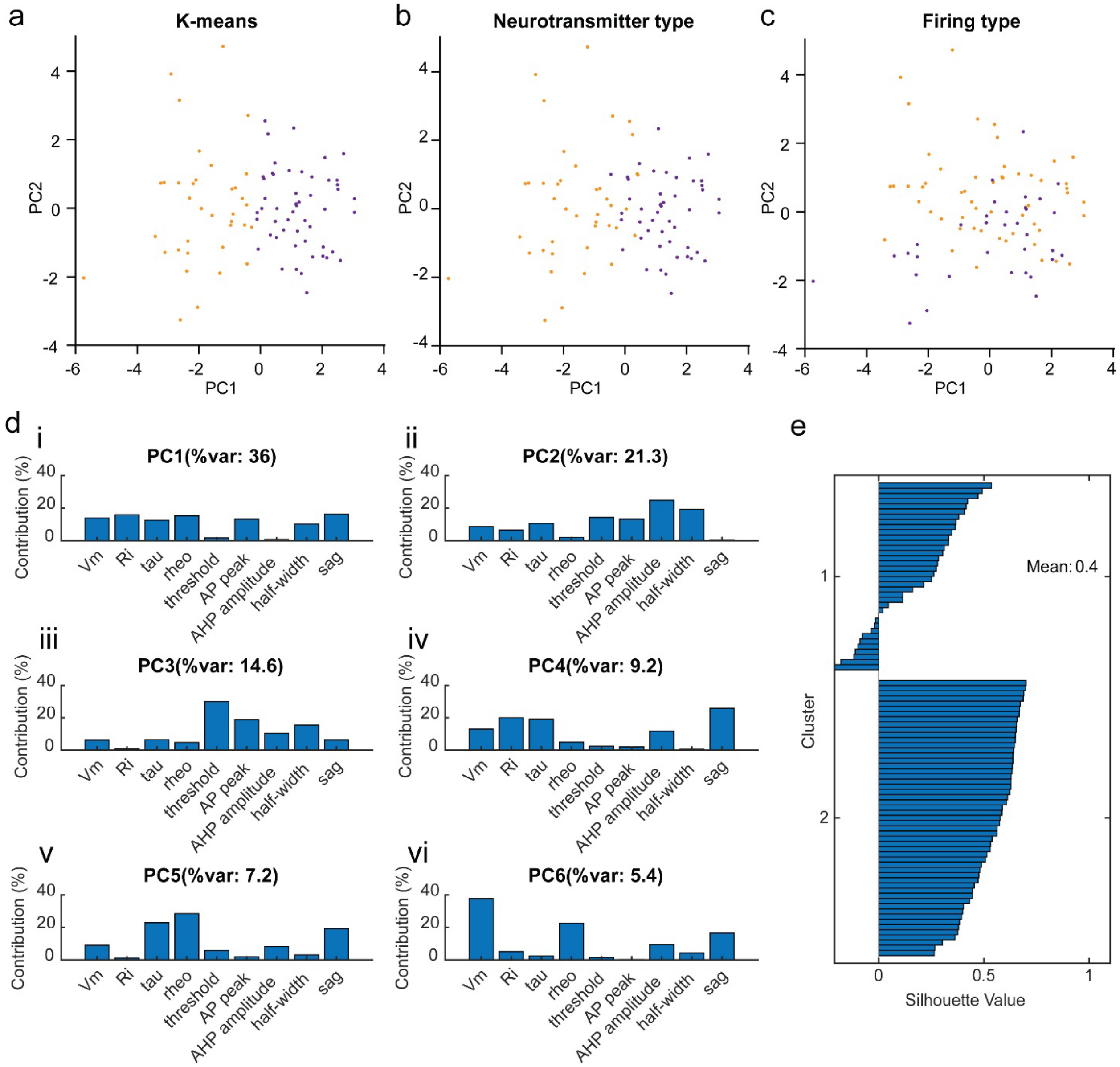

Figure S4: Principal component analysis of electrophysiological parameters. a. Scatter plot for data points represented along the first two orthogonal PC dimensions, color coded by k-means cluster assignment. **b, c.** Same as in **a**, but color coded based on neurotransmitter type **(b,** orange inhibitory**,** purple excitatory**)** or firing type **(c,** orange onset burst, purple multi-spiking**). d.** Percent contribution of z-scored electrophysiological parameters to the first 6 orthogonal PC dimensions that together account for 93.7% of total variance (**i-vi,** PC1-PC6). **e.** Clustering quality assessed by silhouette analysis. Silhouette value for a given point is high if that point is closer to the members of its cluster than the members of the other cluster.

Principal component analysis (PCA) followed by k-means clustering was carried out in MATLAB to assess contributions of cell type on intrinsic electrophysiological parameters. The nine intrinsic parameters included were resting membrane potential, input resistance, membrane time constant, rheobase, AP threshold, AP peak, afterhyperpolarization amplitude, half-width,

and sag amplitude. The data were z-transformed before further analyses. The alternating least squares method handled a few missing entries in PCA analysis. A k-means clustering was performed using an optimal cluster number that was identified for our data using evalclusters() function with the silhouette criterion. Since optimal cluster number was 2, Yule's correlation coefficient (φ) between binary cell types and binary cluster assignments were estimated to assess the overall influence of cell type on the identified clusters. A 2-tailed permutation test at 10,000 iterations shuffled the cell type order relative to cluster while keeping the cell type numbers same in each iteration. From this, a null distribution for the correlation coefficient was generated to compute the p-value of the correlation. This analysis suggested there were 2 groups and that transmitter type explained most of the clustering (Yule's correlation between transmitter type and cluster, φ =-0.85, $p<0.0001$, permutation test for correlation; Yule's correlation between firing type and cluster, φ =0.18, $p=0.14$, permutation test for correlation).

Supplemental Figure S5

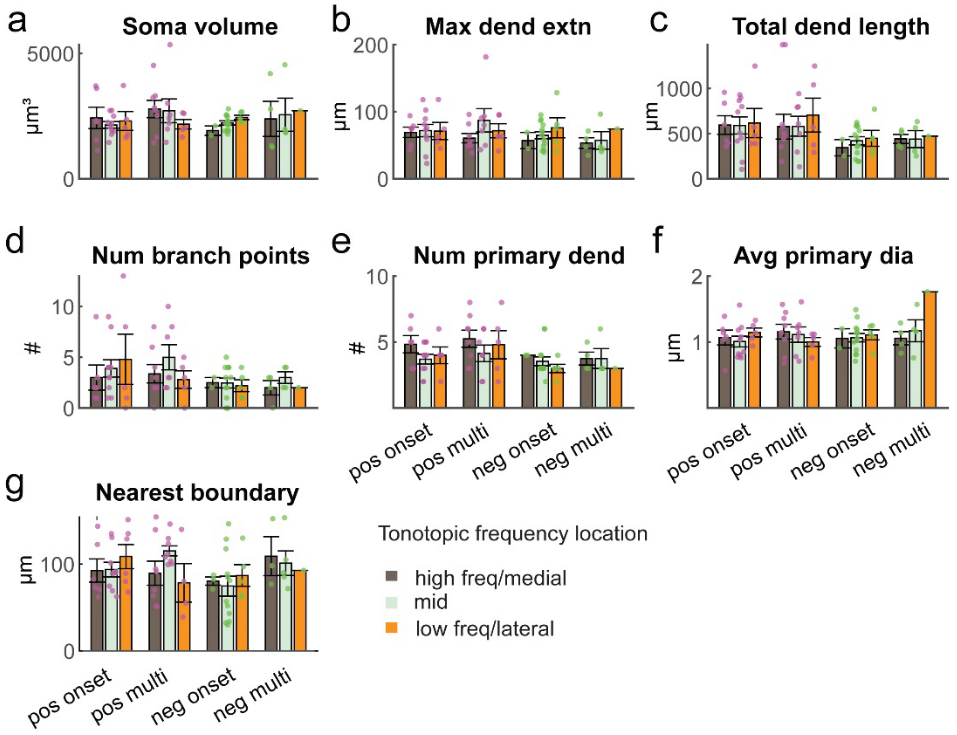

Figure S5: Analysis of morphological parameters and tonotopy. Mean ±SEM of morphological parameters for cell groups broken down by tonotopic locations. Data points in magenta: excitatory cells, green: inhibitory cells. There was only one inhibitory multi-spiking cell in the lateral/low frequency region. **a.** Soma volume. **b**. Maximum extension. **c.** Total dendritic length. **d**. Total number of branch points. **e**. The number of primary dendrites. **f**. Average primary dendrite diameter.

We performed three-way ANOVA with independent variables transmitter type, firing type, and tonotopic location with the dependent variables shown. See tables below.

Table S5: Three-way ANOVA results for morphological parameters with tonotopic location and transmitter type. Analysis of data shown in Figure S5. Bold values are significant at an alpha value of 0.05.

Table S5	Tonotopy				Transmitter x tonotopy				Firing x tonotopy			
	df1	df2	F	p	df1	df2	F	p	df1	df2	F	p
soma volume	2	60	0.0322	0.9684	2	60	0.6181	0.5424	2	60	0.4298	0.6526
max dendritic extn	2	60	0.8571	0.4295	2	60	0.5532	0.5780	2	60	0.4174	0.6606
total dendritic length	2	60	0.3301	0.7201	2	60	0.0165	0.9837	2	60	0.0841	0.9194
n branch points	2	60	0.8945	0.4142	2	60	0.1702	0.8439	2	60	0.9632	0.3875
n primary dendrites	2	60	1.2490	0.2941	2	60	0.8004	0.4539	2	60	0.0608	0.9411
primary dendr dia	2	60	0.5255	0.5940	2	60	1.0257	0.3647	2	60	0.0167	0.9834
nearest boundary	2	60	0.2396	0.7877	2	60	0.4651	0.6303	2	60	1.7885	0.1760

Table S6: Three-way ANOVA results for morphological parameters with transmitter type and firing type. Analysis of data shown in Figure S5. Bold values are significant at an alpha value of 0.05.

Table S6	**Transmitter**				Firing				Transmitter x firing			
	df1	df2	F	p	df1	df2	F	p	df1	df2	F	p
soma volume	1	60	0.0848	0.7719	1	60	1.3396	0.2517	1	60	0.0015	0.9688
max dendritic L	1	60	0.9988	0.3216	1	60	0.1151	0.7356	1	60	0.5660	0.4548
**total dendritic L**	1	60	5.0219	**0.0287**	1	60	0.2064	0.6513	1	60	0.0538	0.8174
**n branch points**	1	60	4.9970	**0.0291**	1	60	0.0886	0.7670	1	60	0.0212	0.8848
**n primary dend**	1	60	5.5875	**0.0213**	1	60	0.5536	0.4598	1	60	0.3434	0.5601
primary dend dia	1	60	0.7720	0.3831	1	60	1.7431	0.1918	1	60	0.7723	0.3830
nearest boundary	1	60	0.5347	0.4675	1	60	0.4398	0.5098	1	60	1.1169	0.2948

Supplemental Figure S6

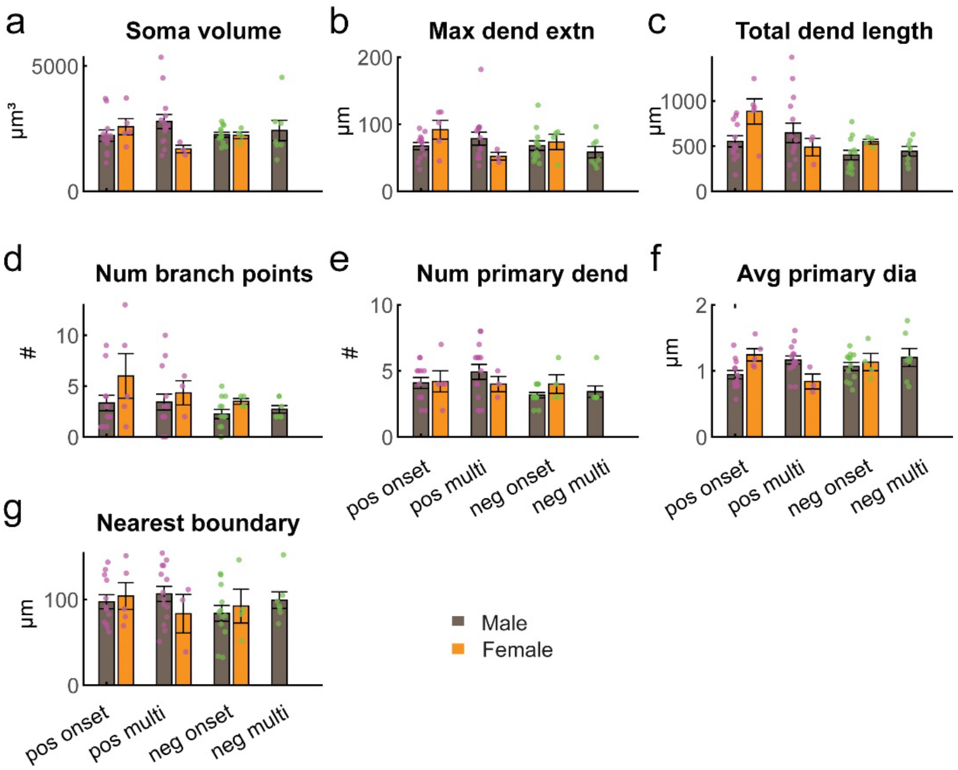

Figure S6: Analysis of morphological parameters and sex. Mean ±SEM of morphological parameters for cell groups broken down by sex. Data points in magenta: excitatory cells, green: inhibitory cells. There were no female inhibitory multi-spiking cells in the dataset. **a.** Soma volume. **b**. Maximum extension. **c.** Total dendritic length. **d**. Total number of branch points. **e**. The number of primary dendrites. **f**. Average primary dendrite diameter.

We performed three-way ANOVA with independent variables transmitter type, firing type, and sex with the dependent variables shown. See tables below.

Table S7: Three-way ANOVA results for morphological parameters with sex and transmitter type. Analysis of data shown in Figure S6. Bold values are significant at an alpha value of 0.05.

Table S7	Sex				Transmitter x sex				**Firing x sex**			
	df1	df2	F	p	df1	df2	F	p	df1	df2	F	p
**soma volume**	1	50	2.6772	0.1081	1	50	0.3711	0.5451	1	50	4.8123	**0.0329**
**max dendritic L**	1	50	0.7991	0.3756	1	50	0.7755	0.3827	1	50	5.0982	**0.0283**
**total dendritic L**	1	50	0.0023	0.9623	1	50	0.7651	0.3859	1	50	4.8965	**0.0315**
n branch points	1	50	0.9606	0.3318	1	50	0.4970	0.4841	1	50	0.6889	0.4105
n primary dend	1	50	0.0053	0.9423	1	50	0.3589	0.5518	1	50	0.6842	0.4121
**primary dend dia**	1	50	1.5213	0.2232	1	50	1.4894	0.2280	1	50	9.4448	**0.0034**

nearest boundary	1	50	0.2869	0.5946	1	50	0.0050	0.9441	1	50	1.2720	0.2648

Table S8: Three-way ANOVA results for morphological parameters with transmitter type and firing type. Analysis of data shown in Figure S6. Bold values are significant at an alpha value of 0.05.

Table S8	**Transmitter**				**Firing**				Transmitter x firing			
	df1	df2	F	p	df1	df2	F	p	df1	df2	F	p
soma volume	1	50	1.1909	0.2804	1	50	1.0817	0.3033	1	50	0.6397	0.4276
**max dendritic L**	1	50	3.0204	0.0884	1	50	4.3717	**0.0416**	1	50	1.5462	0.2195
**total dendritic L**	1	50	6.1260	**0.0168**	1	50	2.3246	0.1336	1	50	0.1029	0.7498
n branch points	1	50	2.3749	0.1296	1	50	0.2907	0.5922	1	50	0.0546	0.8162
n primary dend	1	50	1.8748	0.1770	1	50	0.0022	0.9630	1	50	0.3849	0.5378
primary dend dia	1	50	0.1316	0.7183	1	50	1.5803	0.2146	1	50	0.3384	0.5634
nearest boundary	1	50	0.5294	0.4703	1	50	0.0372	0.8478	1	50	0.1012	0.7517

Supplemental Figure S7

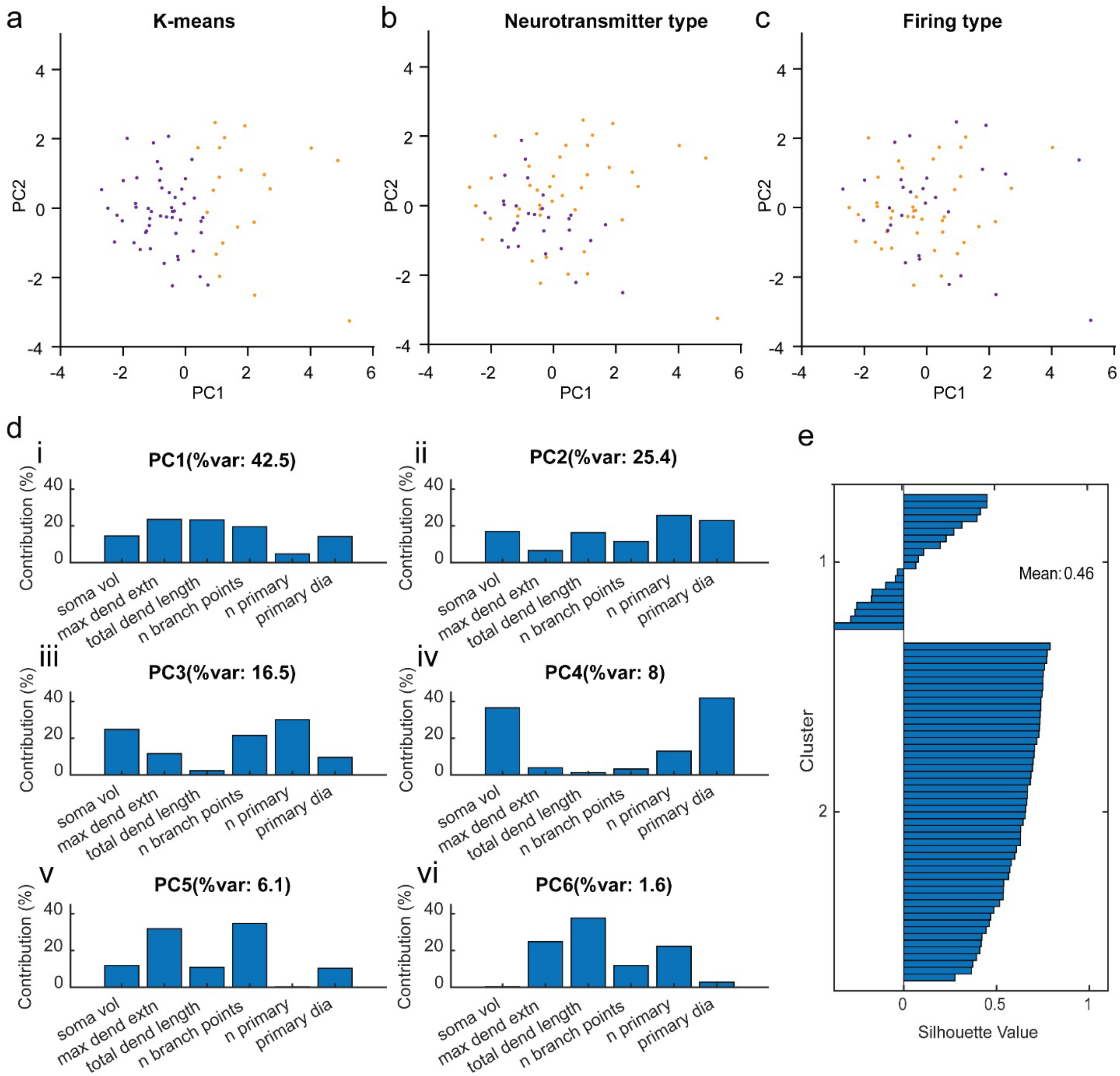

Figure S7: Principal component analysis of morphology. a. Scatter plot for data points represented along the first two orthogonal PC dimensions, color coded by k-means cluster assignment **b, c.** Same as in **a**, but color coded based on neurotransmitter type (**b,** orange inhibitory**,** purple excitatory) or firing type (**c,** orange onset burst, purple multi-spiking). **d.** Percent contribution of z-scored morphological parameters to the first 6 orthogonal PC dimensions that together account for the total variance (**i-vi,** PC1-PC6). **e.** Clustering quality assessed by silhouette analysis. Silhouette value for a given point is high if that point is closer to the members of its cluster than the members of the other cluster.

Principal component analysis (PCA) followed by k-means clustering as in Figure S4 above. The morphological parameters considered were soma volume, average dendritic diameter,

maximum extension, total dendritic length, number of primary dendrites and number of branch points. This analysis again suggested there were 2 groups, and that transmitter type explained the clustering better than firing type (Yule's correlation between transmitter type and cluster, $\varphi=0.34$, $p=0.008$, permutation test for correlation, Yule's correlation between firing type and cluster, $\varphi=-0.05$, $p=0.91$, permutation test for correlation).
