# Supplementary material for: Principal neuron diversity in the murine lateral superior olive supports multiple sound localization strategies and segregation of information in higher processing centers
Source: Commun Biol. 2023 Apr 19;6:432. doi: 10.1038/s42003-023-04802-5 (PMC10115857; doi:10.1038/s42003-023-04802-5)
Supplement: Supplementary file 3 — Description of Additional Supplementary Files [file 42003_2023_4802_MOESM3_ESM.pdf]

Description of Additional Supplementary Files

File name: Supplemental Data 1
Description: Numerical source data.
